# Cell-to-Cell Communication in Learning and Memory: From Neuro- and Glio-Transmission to Information Exchange Mediated by Extracellular Vesicles

**DOI:** 10.3390/ijms21010266

**Published:** 2019-12-30

**Authors:** Gabriella Schiera, Carlo Maria Di Liegro, Italia Di Liegro

**Affiliations:** 1 Department of Biological, Chemical and Pharmaceutical Sciences and Technologies (Dipartimento di Scienze e Tecnologie Biologiche, Chimiche e Farmaceutiche) (STEBICEF), University of Palermo, 90128 Palermo, Italy; gabriella.schiera@unipa.it (G.S.); carlomaria.diliegro@unipa.it (C.M.D.L.); 2 Department of Biomedicine, Neurosciences and Advanced Diagnostics (Dipartimento di Biomedicina, Neuroscienze e Diagnostica avanzata) (Bi.N.D.), University of Palermo, 90127 Palermo, Italy

**Keywords:** extracellular vesicles, learning, memory, synaptic plasticity, tripartite synapsis, tetrapartite synapse, wiring transmission, volume transmission, glial cells, synaptic plasticity

## Abstract

Most aspects of nervous system development and function rely on the continuous crosstalk between neurons and the variegated universe of non-neuronal cells surrounding them. The most extraordinary property of this cellular community is its ability to undergo adaptive modifications in response to environmental cues originating from inside or outside the body. Such ability, known as neuronal plasticity, allows long-lasting modifications of the strength, composition and efficacy of the connections between neurons, which constitutes the biochemical base for learning and memory. Nerve cells communicate with each other through both wiring (synaptic) and volume transmission of signals. It is by now clear that glial cells, and in particular astrocytes, also play critical roles in both modes by releasing different kinds of molecules (e.g., D-serine secreted by astrocytes). On the other hand, neurons produce factors that can regulate the activity of glial cells, including their ability to release regulatory molecules. In the last fifteen years it has been demonstrated that both neurons and glial cells release extracellular vesicles (EVs) of different kinds, both in physiologic and pathological conditions. Here we discuss the possible involvement of EVs in the events underlying learning and memory, in both physiologic and pathological conditions.

## 1. Introduction

At the cellular and molecular level, learning and memory processes are based on the ability of the neural circuits to undergo long-lasting, adaptive modifications of the strength, composition, and efficacy in the connections between neurons, a property known as synaptic plasticity [1,2,3,4,5,6,7,8,9,10,11,12,13]. One of the most significant priming events in the enhancement of synaptic strength is activation of the *N*-methyl-d-aspartate (NMDA) type of glutamate receptors (NMDARs) that transiently elevate post-synaptic concentration of calcium ions. Calcium influx activates calcium–calmodulin-dependent protein kinase II (CaMKII) [14,15] that in turn phosphorylates some target proteins such as kalirin [16], involved in promoting long-lasting modifications of the post-synaptic element through the induction of the expression of further proteins, for example, the α-amino-3-hydroxy-5-methyl-4-isoxazolepropionic acid (AMPA) type of glutamate receptors [16,17]. At the same time, presynaptic voltage-gated calcium channels also play an important role by mediating formation of signaling complexes in the active zones. Presynaptic calcium channels are also regulated by phosphorylation by a variety of protein kinases [15]. It is by now clear that the primary chemical modifications (information) can be retained through an additional chain of secondary biochemical events involving modification of the chromatin structural organization and, consequently, of the expression of specific genes [6,18,19,20]. This “mnemogenic” [21] process should allow later retrieval of memories in response to the appropriate signals.

Synaptic plasticity does not depend only on the activity of nerve cells, but relies on the continuous crosstalk between neurons and the non-neuronal cells around them. Astrocytes, in particular, not only cooperate with neurons at the metabolic level, but also have crucial functions in the formation, maintenance, and potentiation of the neural circuits. By releasing different kinds of molecules (e.g., D-serine, glutamate), indeed, astrocytes are able to influence neuronal transmission [22,23,24]. Like astrocytes, the other glial cells also release molecules that can influence neuronal activity [25]. On the other hand, neurons produce factors that can regulate the activity of glial cells. For example, during brain development myelination can be inhibited by factors released by oligodendrocytes via small, exosome-like vesicles into the extracellular space [26]; such inhibition is later positively counteracted by factors released by neurons [26,27,28].

Notably, in the last two decades, it has been demonstrated that both neurons and glial cells produce and release extracellular vesicles (EVs) of different kinds, both in physiologic and pathological conditions [29,30,31,32,33,34,35]. Moreover, it seems that the molecular mechanisms underlying formation, modification, and potentiation of neural circuits in development, as well as learning and memory processes, rely, at least in part, on the EV-mediated exchange of molecules [28,36].

The crosstalk between neurons and glial cells seems to be altered in most neurodegenerative pathologies; in parallel, production and composition of EVs are modified [37,38,39,40].

EVs are able to cross the blood–brain barrier (BBB) and can be collected from many biologic fluids such as blood, saliva, and urine [41,42,43,44]. Thus, it has been proposed that EVs present in biological fluids can be used as biomarkers of diagnostic value [43,45,46].

Throughout the text we will use the general term EVs for describing all kinds of vesicles; it is, indeed, not easy, up to now, to obtain them as pure preparations and to characterize properly all the subclasses of these membrane-bound structures [47,48]. However, when referring to specific experimental work, for the sake of fidelity to the cited papers, we will use the terms used by the authors.

Here we first summarize some general ideas on the possible involvement of glial cells in transmission, in relation with learning and memory, and then discuss the possible involvement of EVs in the exchange of factors able to regulate these processes. We will also consider how these pathways might be altered in neurologic deficits and whether the ability of EVs to cross the BBB might be exploited to allow transfer of therapeutics to the brain [49]. 

## 2. The Role of Glial Cells in Memory 

For decades astrocytes have been considered fundamental for brain functions because of their metabolic support to neurons and for their ability to regulate the extracellular levels of ions, neurotransmitters, and metabolites, thus maintaining the best conditions for neuronal firing. For example, the brain contains a small amount of glycogen, and this glycogen is found mainly in astrocytes, and is particularly enriched in the hippocampus [50]. This observation is relevant when we consider the role of hippocampus in memory processes [51,52,53].

The dynamic metabolism of astrocytic glycogen and aerobic glycolysis seem to be of the most importance for learning and memory formation [54,55,56,57,58,59]. It has been proposed that glycogen breaks down in astrocytes, giving rise to glucose-1-phosphate, then isomerized to glucose-6-phosphate to be used for glycolysis. Lactate, produced by astrocytes at the end of this metabolic pathway and released in response to neuronal activation, might be taken up by neurons, oxidized to pyruvate and used to synthesize acetyl-CoA, then used in the tricarboxylic acid cycle [60]. The observation that glutamate released from glutamatergic axons is taken up also by the astrocytes which surround the synapses suggested the existence of an astrocyte–neuron lactate shuttle (ANLS), which should be particularly active during the excitatory neurotransmission [61]. Glutamate is the main excitatory neurotransmitter of the brain [62] and its uptake is mediated by a sodium-dependent symport that relies on an increase of the Na^+^/K^+^-ATPase activity. The ANLS model suggests that, by consuming ATP, this process stimulates glycolysis, glucose utilization, and lactate production [61,63,64,65]. Lactate should be then delivered to neurons through different isotypes of the monocarboxylate carriers (MCTs) present in the plasma membranes of both astrocytes and neurons. At the same time, ATP is used by glutamine synthetase (GS) in astrocytes to convert glutamate to glutamine, which is then released to neurons as well [58,66,67]. Notably, insufficient GS activity during synaptogenesis can negatively affect spatial memory in the adult [68]. It is also worth noting that glutamate uptake by astrocytes does not have only a metabolic role, but also limits the glutamatergic spill over responses at extra-synaptic sites [13].

The discovery of a lactate-specific G-protein coupled receptor (GPR81), also known as hydroxyl-carboxylic acid 1 (HCA1) or hydroxyl-carboxylic acid receptor 1 (HCAR1), led to the suggestion that lactate may also, or perhaps mainly, act as an astrocyte-derived hormone, even involved in processes as complex as memory formation and neuroprotection [67,69,70,71,72]. Interestingly, GPR81 and the mRNA encoding it indeed localize to hippocampus, neocortex, and cerebellum [73,74]. In the rat hippocampus the learning process leads to a significant increase in the extracellular lactate levels [75] and inhibition with dichloroacetate (DCA) of lactate production in mice impaired in both learning and memory acquisition [76].

**Figure 1:**
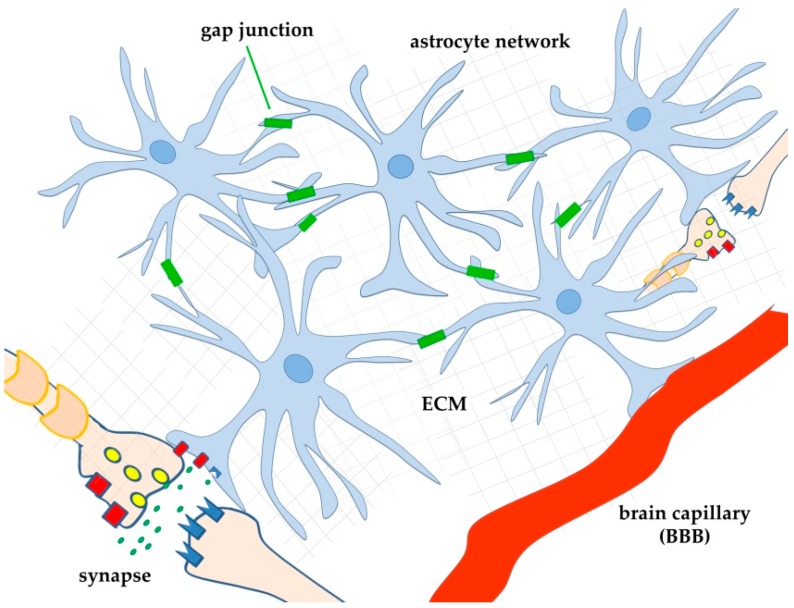
Schematic drawing of astrocyte interconnection by gap junctions (green rectangles). By forming a network, astrocytes can influence synapses far away from the active ones, allowing different (and distant) synapses to interact with each other also in the absence of proximity (lateral regulation of synaptic transmission by astrocytes, [86]). For simplicity, only two distant astrocytes, each interacting with one synapse (model of the tripartite synapse), and one astrocyte interacting with one brain capillary are shown. Thanks to the network, astrocytes can probably also transfer metabolites (for example, glucose) from blood to distant neurons. BBB: blood-brain-barrier; ECM: extracellular matrix.

More recently, it was realized that involvement of astrocytes in neuronal function was even more complex due to their ability to respond to many neurotransmitters and to release a variety of their own signalling molecules (gliotransmitters) [77,78], thus functioning as regulators of neuronal function [79]. To describe this bidirectional exchange of information between astrocytes and neurons the term “tripartite synapse” was proposed [80,81,82,83]. In this model, one astrocyte enwraps one synapse and behaves as an integrated regulator of its activity (Figure 1). Over time, it is becoming increasingly clear that, given its morphology, one astrocyte can actually embrace many synapses [84]. Moreover, astrocytes do not behave as individual cells, as they are connected to each other by gap junctions [85] (Figure 1). These fundamental observations suggested the existence of an even more complex regulatory relationship between neurons and astrocytes, in which astrocytes, by forming a network, can also influence synapses far away from the active ones. Thus, different (and distant) synapses can interact with each other also in the absence of proximity through what has been called “lateral regulation” of synaptic transmission by astrocytes [86]. Calcium waves elicited by neurotransmitters, neuromodulators, and other extracellular cues, in specific microdomains of the astrocyte network, seem to be fundamental for lateral transmission by astrocytes of a variety of molecules that can modulate neuronal transmission, thus also acting on neuronal plasticity, and even on learning and memory [79,83,87,88].

An example of the calcium involvement in astrocyte function is given by strong stimulation of Schaffer collaterals, that elicits glutamate released and activation of CA1 pyramidal neurons. At the same time, it activates inhibitory GABAergic interneurons that release γ-amino-butyric acid (GABA). GABA binds, in turn, to GABA-B receptors on astrocytes, thus stimulating a calcium-dependent release of ATP (a gliotransmitter). In the extracellular environment, ATP is dephosphorylated to adenosine, a signalling molecule that depresses some of the surrounding synapses [86].

Astrocytes express a number of receptors for neurotransmitters, including acetyl-choline, ATP, GABA, endocannabinoids, and glutamate [86,88,89,90,91,92,93]. They also produce neurotransmitters on their own (e.g., glutamate, D-serine, ATP, and GABA) [90,94,95,96,97,98,99] and secrete them through exocytotic mechanisms, not so different from those acting in neurons [100]. This double activity is added to the diversity of neuronal transmission, thus giving rise to a further level of integration in synaptic information and contributing to brain plasticity. For example, it has been recently reported that the astrocytic calcium signalling could also provide a bridge between cholinergic activation and somatosensory plasticity in mouse barrel cortex [90]; moreover, this effect seems to be mediated, at least in part, by D-serine [90].

Combinations of different neurotransmitters can result in different and specific degrees of calcium level modifications. Specificity of calcium responsiveness may in turn influence release of gliotransmitters. It is worth noting that the effect of the gliotransmitters also depends on the neuronal environment [88], as well as on the concentration of specific ions [101]. On the other hand, the degree of astrocyte activity can be influenced by other factors and hormones; for example, astrocytes can be primed to detect cortical network activity by norepinephrine [102]. 

The great variety of functions of astrocytes actually raises a few basic questions: are the astrocytes all identical? Are they all able to release the whole set of the gliotransmitters? Or, like neurons, are they somehow specialized, depending on the brain region and/or the neural circuit in which they work? Although clear answers cannot yet be given, some studies evidenced a certain degree of both region- and circuit-specific diversity [103]. Moreover, it has been proposed that astrocyte heterogeneity can have an effect on brain aging and disease [104]. 

Finally, and surprisingly, it has been recently reported that astrocytic activation is not only necessary for synaptic plasticity, but also sufficient to induce NMDA-dependent long-term potentiation (LTP) in the hippocampus [22,105]. 

## 3. Wiring (Synaptic) and Volume Transmission in the Nervous System

**Figure 2:**
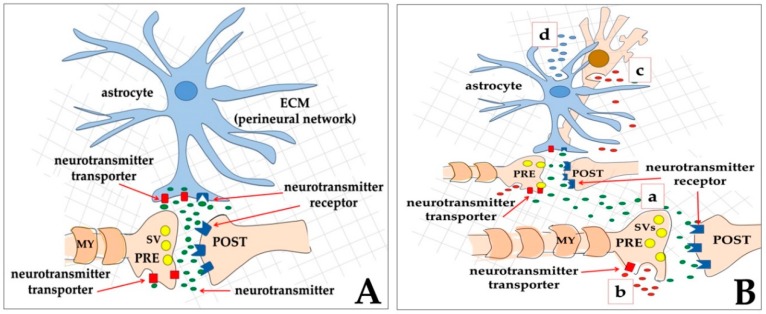
Schematic drawing of two types of neurotransmission, according to the initial proposal of the model [109,110]. (**A**) In the wiring transmission (WT), the presynaptic neuron (PRE) releases neurotransmitter molecules (small green ovals) that bind to their receptors (dark blue) on the post-synaptic element (POST). Excess neurotransmitters are then taken back by the presynaptic neuron through neurotransmitter transporters (red rectangles); In (**B**), a few ways to obtain volume transmission (VT) are represented: (a), the presynaptic neuron (PRE) releases a neurotransmitter (small green ovals) that not only binds to its receptors on the post-synaptic element (POST), but also diffuses at different distances, thus reaching other faraway synapses that will be activated; (b) extrasynaptic release from the axon of signal molecules (small red ovals) into the extracellular matrix, outside the synaptic cleft; (c) extrasynaptic release of signal molecules (small red ovals) from the soma of a neuron; (d) gliotransmitter release from an astrocyte (small blue ovals). In both WT and VT, astrocytes play a central role since they can express on their membranes both neurotransmitter receptors and neurotransmitter transporters, and, in addition, they can release gliotransmitters.

Between the 1980s and the beginning of 1990s, it was realized that the 1-to-1 mode of synaptic transmission was not the only way of inter-neuronal communication, and that extra- or non-synaptic transmission also existed [106,107,108]. The 1-to-1 and the 1-to-many modes of transmission were further defined as wiring transmission (WT) (Figure 2A) that corresponds to both chemical or electric synapses, and volume transmission (VT) (Figure 2B), respectively [109,110,111]. In more detail, indicating with S the source of the signal, and with T the target of the signal, in the WT contact, the ratio (S/T) between the number of S structures and the number of T is S/T = 1, and the communication event is very fast. On the other hand, in VT the ratio is S/T < 1 because the signals produced by a single source may reach more than one target. This latter mode is slow and based on diffusion at a distance of the molecules/ions released by the source. An important aspect of this general model is that VT does not necessarily connect only neurons, but can also involve other cells of the nervous system, such as astrocytes, both as sources and as targets [112,113] (Figure 2). 

**Figure 3:**
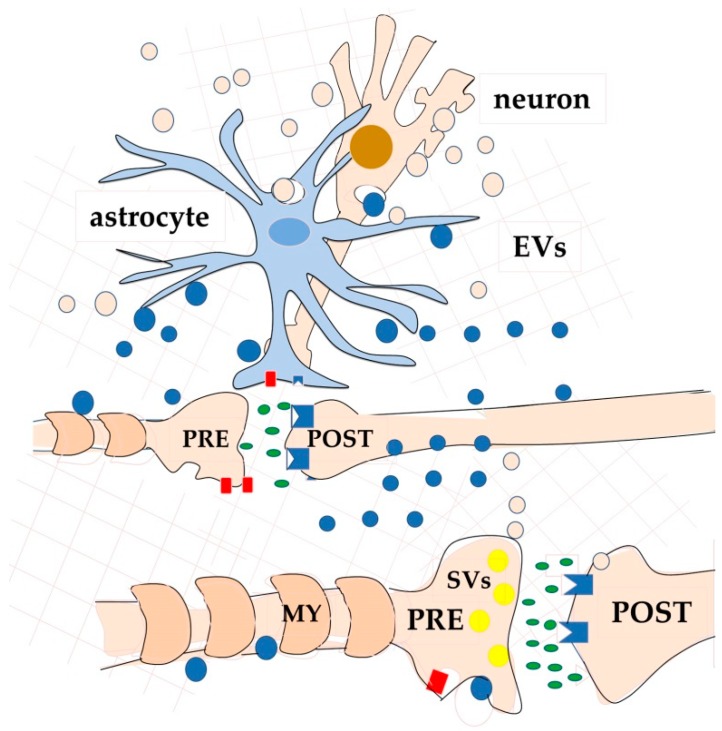
Schematic drawings of one further aspect of VT-neurotransmission [114]. A variety of molecules and metabolites can be exchanged both among neurons, and among neurons and other brain cells, via extracellular vesicles (EVs) of different size and composition. Neurons release EVs (probably for most exosomes) mainly from the soma and dendrites. As an example of a glial cell, an astrocyte is drawn. For clarity, vesicles have been depicted the same colour as the producing cell: light pink produced by the neuron and blue produced by the astrocyte.

The second interesting aspect of VT is that, among the plausible mechanisms of signal release, EVs can be also included (Figure 3) as carriers that allow transfer of packages of signalling molecules such as proteins, microRNAs (miRNAs), mRNAs, lipids, metabolites, etc., to the target cells [28,36,114]. 

It is to be underlined that long-distance VT relies on both the extracellular fluid/matrix (ECF/ECM) and the cerebrospinal fluid (CSF), which indeed contains a variety of neuroactive molecules from small neurotransmitters (e.g., dopamine and noradrenaline) to peptides (e.g., β-endorphin) and proteins [113,115,116]. CSF flows throughout the brain along para-vascular spaces, parallel to the cortical arteries [117,118]. Moreover, ECF and CSF have similar composition, thus suggesting a dynamic exchange of solutes and water between the two compartments, probably through a water-exchanging network that was termed the glymphatic system [119,120]. This fluid network could be responsible for the circulation of EVs, that can be also allowed to cross the BBB, and to appear in the blood. 

## 4. Production of EVs within the Brain and Their Possible Role in Learning and Memory 

### 4.1. EVs in the Nervous System: General Considerations

By now, EVs have been recognized to be not only cellular systems for discarding unwanted components, but also (and mainly) powerful means for intercellular communication, both in prokaryotes and eukaryotes [121,122,123,124,125], and even for trans-kingdom exchange of molecules [126,127], such as in the parasite–host crosstalk [128,129]. EVs are indeed able to transfer proteins, lipids, and nucleic acids that can influence different physiological and pathological functions of the target cells [125,130]. Exchange of vesicles basically permits integration of the activities and responses of the various cells of a given tissue. However, in pathological conditions the same mechanism can promote tumorigenesis as well as the spreading of neurotoxic aggregates [39,131,132,133,134].

On the basis of their origin and composition, EVs have been divided into different classes, the main of which are: microvesicles (MVs, or ectosomes), exosomes, and apoptotic bodies [125,135,136]. Recently, *Caenorhabditis elegans* adult neurons have been also reported to release very large (~4 μm) vesicles (exophers) that contain protein aggregates and organelles [137,138]. MVs vary in size from 100 to 1000 nm and are produced by external budding of the plasma membrane. On the other hand, exosomes are smallest (30–100 nm) and derive from the endosomal system, and in particular from the so called multivesicular bodies (MVBs) [139]. Protein analysis by sodium dodecyl sulphate-polyacrylamide gel electrophoresis (SDS-PAGE) immunoblotting and mass spectrometry evidenced that the EV protein composition varies according to the cells from which it originates. Although MV– and exosome–protein supplies overlap, it has been recently reported that Annexin A1 is typical of MVs [140], while the tetraspanins, once believed to be markers of exosomes, were also found in MVs. In general, however, a certain level of overlapping both in size and composition among the different classes of vesicles has been described; thus, it is not easy to use a specific nomenclature only on the basis of size and/or composition of the vesicles [48,124].

It is worth remembering that the brain cell activity as well as its effects are influenced by the surrounding environment, which includes a variety of cells, but also the molecules of the ECM, produced by the cells themselves. The complex of these components form the perineural net (PNN) [141]. A reduction in the expression of ECM components can affect synaptic plasticity, but an increase in ECM activity (for example, as a result of pathologic astrogliosis) can also be harmful [141].

**Figure 4:**
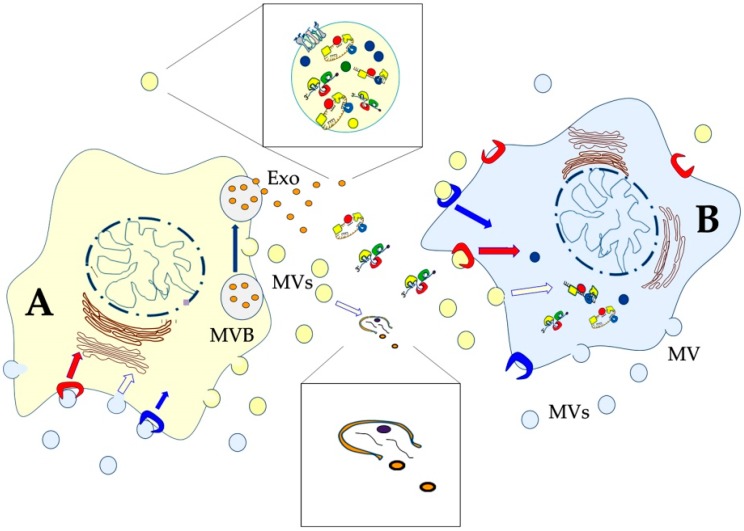
Schematic drawing of two cells that communicate with each other by exchanging extracellular vesicles of different sizes and origins. Some vesicles directly bud from the plasma membrane (microvesicles, MVs), while exosomes (Exo) derive from the multivesicular body (MVB). For clarity, vesicles have been depicted in the same colour as the producing cell: yellow produced by the yellow cell (A), and light blue produced by the light blue cell (B). After release, some EVs are quickly lysed and release their content into the extracellular space (lower enlarged view). Some of them contain matrix metalloproteases and other hydrolytic enzymes responsible for the digestion of various ECM components. Alternatively, intact EVs can interact with the target cells that can internalize them through a variety of pathways [145]. The vesicles can also directly fuse with the plasma membrane of the target cell. Both MVs and exosomes are endowed with proteins, lipids, and nucleic acids that can influence different physiological and pathological functions of the target cell (upper enlarged view).

In comparison to other tissues, brain ECM contains higher amounts of hyaluronic acid (HA), thrombospondin, and proteoglycans, including aggrecan, neurocan, and versican; these molecules serve as ligands for receptors (integrins) present in the plasma membranes of brain cells. ECM around the synaptic cleft has a special composition in which tenascin, thrombospondin, and pentraxins dominate [141]. In addition to these general properties, specific compositions of given brain areas have been described. For example, it has been reported that PNN in the mouse sensory cortex is enriched in aggrecan and glycosylated aggrecan [142]. On the other hand, HA is present in PNNs throughout the mouse cortex, but with region-dependent differences [143]. Most important, composition of the ECM can be modified through the action of matrix metalloproteases (MMPs) and serine proteases [141]. Notably, proteases as well as glycosidases have been identified in EVs, sometimes on their surface [144], thus suggesting that brain cells can modify the ECM by releasing EVs into it. After leaving the producing cell, indeed, many EVs are quickly lysed (Figure 4) and release their content into the extracellular space [145], thus mediating digestion of various ECM components. These events could both turn in favour of the invasive capacity of tumour cells [146] or positively act on synaptic plasticity [147]. Alternatively, intact EVs can interact with the target cells that can internalize them through a variety of endocytic pathways, including clathrin-dependent endocytosis, clathrin-independent pathways (e.g., caveolin-mediated uptake), macropinocytosis, phagocytosis, and lipid raft-mediated internalization. Finally, the vesicles can directly fuse with the plasma membrane of their target cells [145,148,149] (Figure 4). Thus, in most cases, EVs release their content into the cytoplasm of the recipient cell (horizontal transfer). Alternatively, they can be released again into the extracellular space, following the fusion of the endosome with the plasma membrane (transcytosis) [150]. 

In the central nervous system (CNS) neurons seem to internalize exosomes mainly through endocytosis or phagocytosis [27,151], while microglial cells seem to use macropinocytosis [152]. Interestingly, in a microfluidics culture system it has been recently confirmed that some exosomes internalized into neurons are re-secreted by a mechanism consistent with the hijacking of secretory endosomes by the exogenous exosomes [134].

Both normal and pathological EVs can transfer proteins such as receptors and ligands. For example, oligodendroglioma cells in culture have been found to transfer pro-apoptotic proteins (such as the Fas ligand, FasL, and the tumour necrosis factor (TNF)-related apoptosis-inducing ligand, TRAIL) to astrocytes and neurons, thus inducing in these cells an increase in the frequency of apoptosis [153,154].

The lipid components of EVs, important for providing structural stability, but probably also for allowing formation, release, and uptake of the vesicles, have been also analysed; from these studies an enrichment in cholesterol, sphingomyelins, glycosphingolipids, and phosphatidylserine (PS), as well as in the monosialoganglioside GM3 was observed both in exosomes and MVs [155,156,157,158].

In addition to proteins and lipids, most EVs also transport nucleic acids. In particular, EV-mediated transfer of mRNAs, long (>200 nt) non-coding RNAs (lncRNAs), and miRNAs (19–23 nucleotide non-coding RNA sequences), has been proposed as a new form of intercellular communication through which cells can influence gene expression of their neighbours at the epigenetic level [33,159].

**Figure 5:**
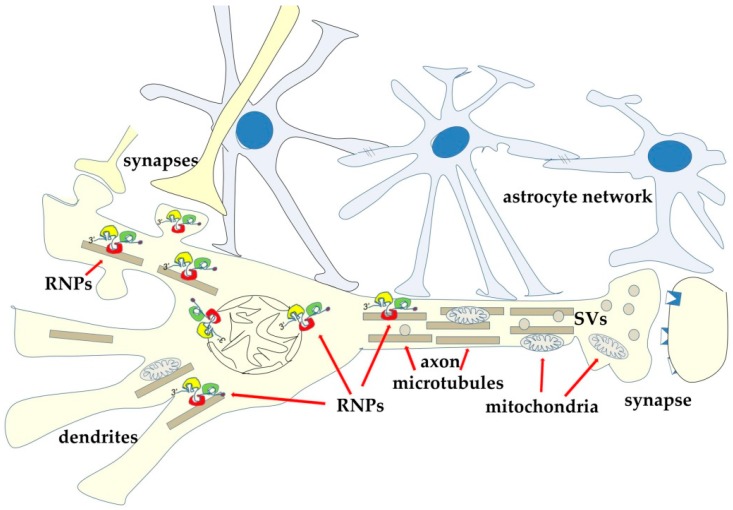
Neurons are highly polarized cells with only one nucleus and highly differentiated peripheries. Polarization depends on the cytoskeleton-dependent trafficking of organelles and vesicle/molecule complexes in both anterograde- and retrograde-direction. Among the transported complexes, ribonucleoprotein complexes (RNPs) have been also described. RNPs contain a variety of RNAs and RNA-binding regulatory proteins (RBPs). During their trip to the periphery mRNAs are repressed. It has been reported that, at post-synaptic sites, upon synapse activation, and largely in response to calcium waves, some RBPs undergo post-translational modifications that allow the release and translation of mRNAs. Some of the newly synthesized proteins can accumulate at the synapse, while others can shuttle back to the nucleus to modify chromatin structure and expression. At the same time, microtubules ensure transport of organelles such as mitochondria and synaptic vesicles.

Together with these different classes of RNA, EVs also contain RNA-binding proteins (RBPs) [160,161]. Given the known role of these latter proteins in the sub-cellular RNA localization, it has been suggested that during vesicle biogenesis RBPs can regulate accumulation of selected RNAs into vesicles [161,162,163]. In the cells, RBPs are involved in the post-transcriptional regulation of gene expression, thanks to their ability to regulate maturation and trafficking of the different classes of RNA to which they bind [164,165,166]. This activity is of particular interest in neurons, where different species of RNA are transported from the nucleus to the peripheries of the cells (Figure 5). Neurons are, indeed, the most polarized cells in the body and obtain a motley distribution of proteins and organelles thanks to a variety of mechanisms, all of which require a functional cytoskeleton (Figure 5). The bidirectional trafficking of organelles and molecules along both dendrites and axons involves the microtubules and the microtubule-associated motors that drive anterograde (kinesins) and retrograde (dyneins) ATP-dependent traffic. In the periphery, such as in the dendritic spines, cargoes can be transferred to actin microfilaments and to the corresponding myosin motors [167].

Recent studies based on live imaging experiments in *Drosophila* larvae and mouse hippocampal neurons show that synaptic vesicles and proteins of the presynaptic active zone (AZ) are co-transported along the axons by forming structures that have been defined presynaptic lysosome-related vesicles (PLVs) [168]. Loss of the lysosomal kinesin adaptor ADP-ribosylation factor-like protein 8 (Arl8) causes accumulation of both synaptic vesicles and AZ-protein-containing vesicles in the neuronal body, with the consequent alteration of the presynaptic structures and impairment of neurotransmission [168]. Super-resolution microscopy and live imaging have also revealed the existence of previously unknown actin-based structures, interacting with microtubules [169].

Reorganization of the neuronal cytoskeleton is critical in learning and memory processes: for example, it was found that theta burst stimulation produced a significant increase in the number of dendritic spines and a concentration of polymerized actin in the targeted dendrites [170,171]. Integrity of microtubules has been also recognized as essential for memory (for example: [172]). Given their highly charged surfaces, both microtubules and microfilaments can also bind counterions and conduct electric signals [173].

It has been known for over 10 years that both astrocytes and neurons release EVs [31,32], and that this way of cellular communication is involved both in development and adult functioning of the CNS [28,130]. For example, EVs produced by neurons and astrocytes contain two angiogenic factors: fibroblast growth factor 2 (FGF2) and vascular endothelial growth factor 2 (VEGF2) [31,32]; thus, they are probably, at least in part, responsible for inducing the brain capillary endothelial cells (BCECs) to form the BBB. Interestingly, in a co-culture in vitro system, the effects of neurons and astrocytes on the BCEC ability to acquire a BBB phenotype are synergic [174].

EVs released by neurons also regulate their own synaptic activity: after depolarization, cortical neurons are indeed able to release proteins such as, for example, the L1 cell adhesion molecule (L1CAM), the glycosylphosphatidyl-inositol (GPI)-anchored prion protein, and the glutamate receptor subunit GluR2/3, through EVs [30]. On the other hand, it has been reported that, in differentiated neurons, the synaptic AMPA and NMDA glutamatergic receptors modulate the release of exosomes at the synapses [175,176]. Thus EVs can act as new routes for trans-synaptic communication [28,177]. It has been found, for example, that, at the *Drosophila* larval neuromuscular junction, the EV-dependent, presynaptic release of the Wnt-binding protein Evenness Interrupted/Wntless/Sprinter (Evi/Wls/Srt) is fundamental for postsynaptic Wnt signal transduction [178]. 

Astrocytes release both MVs and exosomes. MV shedding depends on activation of P2X7 purinoceptors and of acid sphingomyelinase (SMase). SMase is transferred to the plasma membrane outer leaflet, where it modifies the membrane structure/fluidity leading to vesicle shedding [179]. Interestingly, astrocytic exosomes have been reported to contain mitochondrial DNA [180]. EVs released by astrocytes also contain matrix metalloproteinases involved in extracellular matrix remodelling [181], as well as neuroprotective factors such as heat shock protein 70 (Hsp70)/heat shock cognate protein 70 (Hsc70) and synapsin I, which are also able to promote neurite outgrowth [182,183]. Recently, it has been shown that EVs released by astrocytes contain apolipoprotein D (apo D) that, upon internalization by neurons, contributes to their oxidative stress response [184]. Notably, the astrocyte EV cargo can be different depending on the external conditions: when stimulated with a trophic (ATP) or with an anti-inflammatory stimulus (interleukine 10, IL10), rat primary astrocytes release vesicles containing proteins involved in increasing neurite outgrowth, dendritic branching, and synaptic transmission, as well as in promoting neuronal survival [185]. On the other hand, when astrocytes are stimulated with IL-1β (inflammatory stimulus), their EVs contain proteins that could stimulate peripheral immune response [185]. 

Like astrocytes, the other glial cells are also able to release EVs. Oligodendrocytes constitute a functional unit with neuronal axons [186] and secrete vesicles containing proteins and myelin lipids [187] in a calcium-dependent manner, in response to glutamate released by neurons. Oligodendrocyte EV cargoes are, in turn, internalized by neurons, and regulate their metabolism, also exerting neuroprotection [27]. During CNS development, once the neurons have reached their destination and established their contacts, the role of oligodendrocyte–neuron communication is even more important. In that period there is an intense vesiculation activity that will allow formation of the myelin coating. EVs released from oligodendrocytes contain factors that inhibit myelination, while neuronal EVs will later stimulate oligodendrocytes to activate it [26,27]. During this process, the role of astrocytic EVs is also fundamental: by delivering synapsin 1 protein, they stimulate neuronal vitality and allow formation of new synapses [183]. At the same time, it is important to remember that initiation of myelination by oligodendrocytes is also stimulated by neuronal impulses, that is, by the release of glutamate from electrically active axons themselves. Synapse activity can even induce translation of the myelin basic protein (MBP), one of the major proteins in myelin [188]. 

Microglia release EVs too, and these EVs not only contribute to regulation of the inflammatory response [189], but also participate in regulation of synaptic activity [190]; for example, they expose on their membrane the active *N*-arachidonoyl-ethanolamine (AEA) endocannabinoid, which can inhibit presynaptic transmission in GABAergic neurons [191]. Interestingly, secretion of exosomes from microglia is stimulated by serotonin [192], a neurotransmitter involved in learning and memory [193,194]. This finding suggests that exosome release from these cells might be impaired in brain pathologies involving alterations of serotonin-dependent neurotransmission.

Exosomes released by neurons have been found to be internalized by microglia that is thus stimulated to remove damaged neurites [195]. 

Finally, as central components of the BBB, BCECs also release EVs. By mass spectrometry (MS)-based shotgun proteomics it was found that, after stimulation with the proinflammatory cytokine TNF, EVs from BCECs contained active proteins and transcription factors that could propagate inflammation across the BBB and within the brain [196].

**Figure 6:**
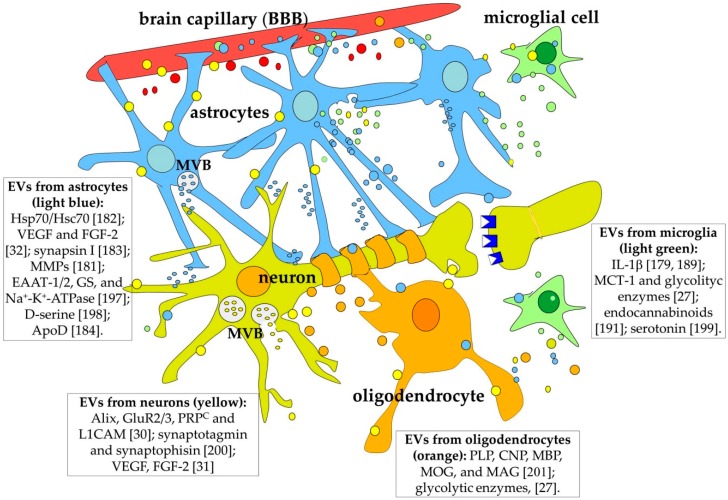
All the cell types in the CNS release EVs of different size, origin, and composition. For clarity, both larger and smaller vesicles have been coloured the same colour as the producing cell. A few components of the vesicles are reported in the inserts with the relevant references [27,31,179,182,183,184,189,197,198,199,200,201]. Abbreviations: ApoD, apoprotein D; BBB, blood–brain barrier; CNP, 2′,3′-Cyclic Nucleotide 3′ Phosphodiesterase; EAAT, excitatory amino acid transporter; FGF, fibroblast growth factor; GS, glutamine synthetase; MAG, myelin-associated glycoprotein; MBP, myelin basic protein; MCT1, monocarboxylate transporter 1; MMPs, matrix metallopeptidases; MOG, myelin oligodendrocyte glycoprotein; MVB: multivescicular body; PLP, myelin proteolipid protein; PRP^C^, cellular prion protein; VEGF, vascular endothelial growth factor.

To summarize, EVs released by all the classes of brain cells can play a role of central importance in the CNS as carriers of regulatory signals, some of which are probably involved in neuronal plasticity, learning, and memory (Figure 6). In the next paragraph, we will discuss evidence suggesting that this is indeed a possibility.

### 4.2. Synaptic Plasticity: The Possible Role of EVs and Their Cargoes

As discussed in the previous paragraph, all the brain cell types are able to produce and release different kinds of EVs. Moreover, both neurons and glial cells release molecules that affect neuronal plasticity both in development and in the adult [28]. Thus, we can suppose that at least some of the learning/memory regulating molecules are secreted via EVs. An observation in favour of this hypothesis is that EV production in the brain is, at least in neurons, regulated by synaptic glutamatergic activity and calcium influx through NMDAR and AMPA receptors in the post-synaptic elements [136,175]. In agreement with this observation, MVBs seem to be much more concentrated in neuronal soma and dendrites than in axons [136] (Figure 6).

Notably, astrocytes are probably the glial cells that give the highest contribution to neuronal plasticity because of their ability to release gliotransmitters such as D-serine [96,202]. It is probable that these factors are transferred at least in part by EVs. D-serine, for example, was evidenced by high performance liquid chromatography (HPLC) in exosomes purified from human serum [198]. Similarly, astrocytic EVs contain functional excitatory amino acid transporters (EAAT)-1 and -2, fundamental for glutamate homeostasis, together with Na^+^/K^+^-dependent ATPase and glutamine synthetase (GS) [197] (Figure 6). As mentioned, they also contain Hsp70/Hsc70 and synapsin I, which are also able to promote neurite outgrowth [182,183]. The latter protein, in particular, has an important role in presynaptic traffic of synaptic vesicles, and it has been reported that a lack of synapsin I induces a lessening of associative memory strength [203].

Mnemogenic processes, however, are not based only on transient modification of preexisting proteins. They indeed rely on long-lasting modifications of chromatin structure and gene expression. Interestingly, the most important property of EVs in all the cell systems studied up to now seems to be their ability to influence the receiving cells at the level of gene expression through a variety of mechanisms, all of which can be considered epigenetic in nature [33,204,205,206]. As mentioned, EVs contain proteins, mRNAs, long non-coding RNAs and miRNAs, all of which can affect the genetic activity of the recipient cells [207]. From this point of view, it is quite intriguing to find that astrocytes, for example, secrete exosomes enriched in miRNA species barely visible in the astrocytes themselves [208]. It is, however, still unclear whether all these miRNAs are present in all the astrocyte-derived exosomes; it is possible, indeed, that different astrocytes release exosomes with different composition. Moreover, different exosome populations might have different targets such as specific neurons, oligodendrocytes, and other astrocytes. In any case, among the astrocytic miRNAs identified in exosomes till now, some are suggested to be involved in neural plasticity [209]. miRNA-26a, for example, has a variety of target mRNAs, among which those encoding the glycogen synthase kinase 3 beta (GSK-3β) [210], phosphatase and tensin homolog (PTEN) [211], and the brain-derived neurotrophic factor (BDNF) [212] all have a role in neurite outgrowth. 

Interestingly, genes that encode miRNAs often form clusters transcribed as polycistrons, which contain 3–6 miRNAs; these latter molecules tend to target functionally related mRNAs [209]. One of these clusters is miR-17-92—five out of the six members of this cluster target and downregulate PTEN, a tumour suppressor protein that also inhibits axonal elongation [213]. The importance of the cluster in brain function has been recently confirmed: its ablation in mouse, indeed, significantly impaired hippocampal-dependent learning and memory, and, in particular, social recognition memory, novel object recognition, and Morris water-maze tests [214]. All the miRNAs of the cluster have been found in astrocytes and at least one of them (miR-19a) has been found in exosomes [209,215]. A further miRNA that targets PTEN and is also found in astrocytic exosomes is miR-26a [211]. Another interesting case involves miR-124, that targets Enhancer of zeste homolog 2 (EZH2), a histone-lysine *N*-methyltransferase enzyme, that methylates histones and represses transcription; miR-124 is involved in neuronal differentiation [216]; it is the most intensively expressed miRNA in the brain and has been found in the serum exosomes of acute ischemic stroke patients [217].

In addition to those cited above, other miRNAs have been also suggested to play a role in neural plasticity (Table 1); for most of them the presence in circulating EVs, at least in some conditions, has been also reported (Table 1, last column).

The exciting finding that also ribosomes may be horizontally delivered from oligodendrocytes (Schwann cells, in the peripheral nervous system) to axons has been also reported [218,219,220]. This observation suggests that glial cells may contribute, at least in some conditions (e.g., demyelination), to local axonal protein synthesis by supplying mRNAs and protein synthetic machinery [220].

Notably it has been reported that exosomes produced in the periphery under conditions of environmental enrichment, mainly by circulating immune cells, contain miR-219 and can increase physical/intellectual activity, as well as learning and memory in rats. Moreover, they seem able to stimulate oligodendrocyte precursor (OPC) differentiation into myelinating cells [221]. This finding adds further complexity to the EV-mediated cell-to-cell communication related to synaptic plasticity and memory because it suggests that some signal can also come from the periphery, particularly under special conditions such as physical activity [222,223]. Of similar importance is the evidence that exosomes derived from neuronal cell lines can induce mesenchymal stem cells (MSCs) to develop neuron-like morphology and to express neuronal-specific genes [224]. In particular, by microarray analysis, it was found that these exosomes contained high amounts of miR-125b (already known to play a role in neurogenesis) [224]. By the way, neural stem progenitor cells (NSPCs) were found to release EVs too, both in proliferating and differentiating conditions; however, EVs derived from differentiating NSPCs induce differentiation in a dose-dependent manner, and in a cell-type specific direction—EVs from astrocyte-like cells can indeed drive NSPC differentiation toward glial lineage [225].

Although all these data on miRNAs are highly promising, more studies are required to better understand how and when miRNAs are sorted to EVs of the different brain cells, and how and in which conditions they can affect neural plasticity.

In summary, all brain cells are able to produce and exchange molecules that can have a crucial role in neuronal plasticity and in long-term modifications of synaptic strength. Much evidence suggests that these cell-to-cell communications rely, at least in part, on different classes of EVs.

## 5. EVs in Neuropathology

In recent years it is becoming increasingly clear that the cargoes of EVs of brain origin can be different in pathological conditions such as neurodegenerative and psychiatric diseases.

The CNS is obviously difficult to reach for direct analysis, and the investigation of its in vivo conditions is based mainly on the analysis of peripheral markers, even though, due also to the presence of the BBB, the odd relationship between peripheral and central conditions often limits the significance of these studies. The discovering of EVs and the description of their unique characteristics is giving researchers new tools for studying CNS pathologies.

In this context, an important observation is that EVs derived from individuals affected by distinct pathologies, or from different cell types of an affected organism, present specific cargoes. 

One peculiar quality of the EVs, and in particular of the exosomes, is their capacity to cross the BBB, so that the analysis of neural-derived exosomes in plasma can give information on CNS status [149,250,251] and might provide biomarkers for different brain pathologies, including mental disorders [136]. On the other hand, it has been suggested that EVs themselves may contribute to alter the integrity of the BBB and to spread diseases and neuroinflammation [252]. Under pathological conditions, they can indeed contain toxic proteins such as prions [131,253], amyloid peptides [132], hyper-phosphorylated Tau protein [39,134], and α-synuclein [133,254,255].

The most studied pathology among those affecting human brain capacity of learning and memory is Alzheimer’s disease (AD). AD, considered the main cause of dementia, is characterized by cerebral deposition of β-amyloid (Aβ) plaques and by the formation inside neurons of neurofibrillary tangles containing hyper-phosphorylated tau protein. The tangles also contain small pieces of microtubules, and indeed, due to the loss of function of the tau protein, the microtubule dynamics and function are impaired in both anterograde and retrograde trafficking along the axons of molecules/complexes/organelles. As a consequence, in a process lasting many years, cortical neurons and synapses are lost in brain areas involved in memory and learning, especially the prefrontal cortex and hippocampus [256,257]. Eventually β-amyloid plaques and neurofibrillary tangles may also extend to other brain formations [258] such as limbic and association areas [259]. As the disease progresses, aggregates of Aβ are also found in motor areas, CSF, and even in regions not directly belonging to brain, such as the eyes [257]. 

EVs are suspected to be directly involved in the progression of AD due to their ability to transport and spread Aβ and tau structures (recently termed “tauons” [260]). In addition, exosomes have been found in close association with amyloid plaques and hyper-phosphorylated tau tangles [132,261]. The connection between EVs and the dissemination of infecting particles is reinforced by the finding that the Aβ and tau oligomers follow the same route as the EVs, and, inside the cells, they are mostly localized in late endosomal, lysosomal, and MVB compartments [262,263].

Autophagy is directly connected with the production and elimination of Aβ. In neurons Aβ is a short peptide deriving from the processing of the membrane beta amyloid precursor protein (APP) by the β-and γ-secretases enzymes [264,265]. In AD, the autophagosomes are not readily converted in autophagolysosomes, causing an accumulation of the autophagic vacuoles containing Aβ in the axons of the affected cells [264]. This eventually leads to the release from neurons of a great amount of Aβ-containing exosomes in the extracellular space [266]. In this regard, it has been suggested that autophagy and exosome production and release might share not only their route but probably also their molecular machinery [267].

In addition to the markers specifically identified as AD signatures, many studies evidenced that the levels of a variety of miRNAs are altered in the circulating exosomes of AD patients [46,268]. For example, miR-139 is overexpressed in AD, and its increase reduces the expression of the cannabinoid receptor type 2 (CB2) [247]. CB2 is a membrane protein present on activated microglial cells and involved in synaptic plasticity, and in the regulation of memory formation as well [269]. Thus, alteration of miR-139 impairs hippocampal function, and affects learning and memory acquisition.

Among other molecules, in neural-derived exosomes extracted from the AD patients’ blood, an increase of insulin resistance molecular markers, such as phosphorylated forms of insulin receptor substrate 1 (IRS-1), has been also noticed [250,270]. The levels of these markers were associated with the worsening of brain atrophy [271]. Interestingly, modifications in the concentration of markers of insulin resistance were also found in EVs derived from the plasma of patients suffering schizophrenia. By magnetic resonance spectroscopy an increase of glucose concentration in the brain of these individuals was noticed [272]. Moreover, it was shown that the increase of insulin resistance and of glucose concentration are related to memory deficit [272].

Actually, one possible cause of AD development is the failing of blood supply to the brain because of reduced circulation. The use of the PRotein Organic Solvent PRecipitation (PROSPR) technique [273] allowed researchers to find that hypo-perfused mice showed characteristics resembling those of AD patients [273].

All these studies raise the intriguing possibility that in the nervous system-derived EVs both molecules able to protect the brain from damage and molecules spreading the pathological condition can be found, thus confirming the idea that EVs can act in AD as a doubled-edged sword [263]. For example, it has been recently found that traumatic brain injury (TBI) induces in the rat hippocampus a significant increase of phosphorylated astrocytic gap junction protein connexin 43 (Cx43). This latter event seems to cause a rapid diffusion of damage signals throughout the astrocytic network, with a secondary spreading of cell death also to brain regions not affected by the initial trauma: astrocytic activation should be therefore negative for the brain. At the same time, and in correlation with Cx43 phosphorylation, however, TBI promotes exosome release, and treatment with exosomes could partially restore LTP after TBI [274]. This finding confirms that at least the EV-mediated astrocyte response to traumatic events should be in principle neuroprotective, even if the further spreading of cell death signals across the gap junctions can then increase damage.

Similar to AD neurons, dopaminergic neurons affected by Parkinson’s disease (PD) overexpress a protein (in this case, α-synuclein) that, in deregulated conditions, tends to form aggregates (Lewy bodies) [275]. PD neurons release exosomes that are transferred to healthy cells, causing in them alterations in the endosomal sorting complexes required for transport (ESCRT), and causing, in turn, an increased production of exosomes loaded with α-synuclein [133,254,255].

In this context of neuro-degeneration, it is also worth noting that, even though most of the molecules implicated in the development of dementia are still unknown, one compound strongly suspected to be at least in part involved in the failing of memory circuits in older individuals is the very long chain C24:1 ceramide [276]. C24:1 ceramide is present in EVs obtained from the sera of adult humans of any age, but its amount progressively increases in older people. It has been proposed that circulating EVs, loaded with increasing amounts of very long chain ceramide, could contribute to cell aging and memory impairment in older adults [277].

As for AD, an increased concentration of specific miRNAs can be characteristic of given pathologies. For instance, exosomes found in frozen post-mortem prefrontal cortexes contain high amounts of miR-497 and miR-29c, respectively, in schizophrenia patients and in bipolar individuals [235], thus suggesting that EVs can offer an important diagnostic tool also in the case of psychiatric disorders.

In summary, brain EVs contain an entire supply of molecules that change depending on different metabolic and pathological states of the brain cells. Thus they seem to represent a promising blood fraction for the easy detection of biomarkers related to specific brain damage and dysfunctions (see below) [278].

## 6. EVs in Diagnosis and Therapy of Memory Deficits

**Figure 7:**
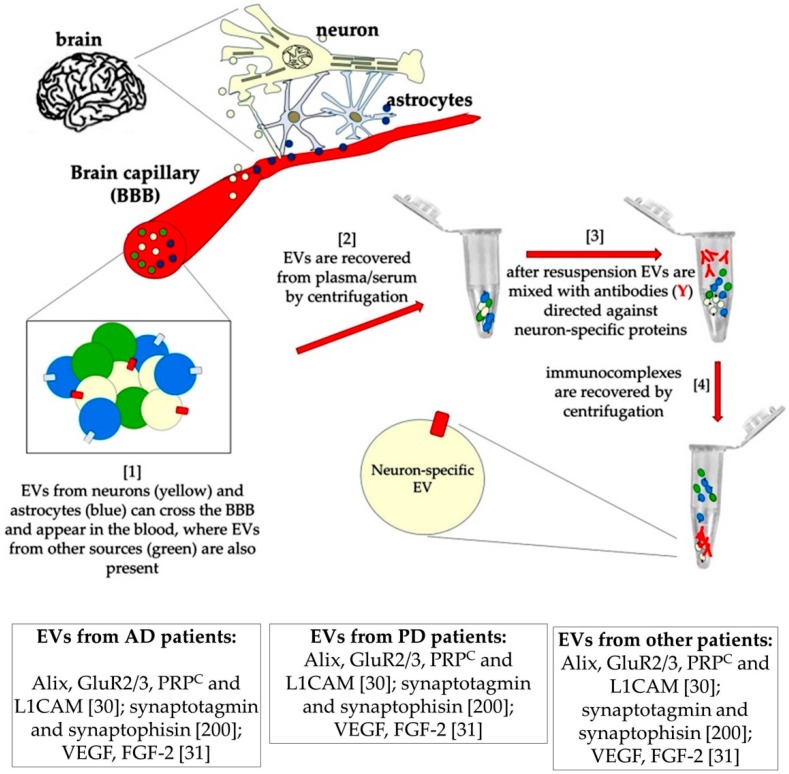
Schematic drawing of a procedure for purifying neuron-derived EVs, according to [250]. A few molecules that have been found increased (upward arrows) or decreased (downward arrows) in EVs purified from the blood of patients are reported in the boxes, together with some relevant references [30,31,200].

As reported above, EVs are able to cross the BBB and can thus be recovered in plasma (Figure 7). These properties have suggested that EVs and their composition might be used as biomarkers of brain conditions. At the same time the possibility has been explored to use them as carriers for delivering therapeutic molecules to the brain.

### 6.1. Circulating EVs as Biomarkers

An interesting example of diagnostic observations based on EVs is offered by the neurotrophin BDNF. Like many other regulatory factors, BDNF is synthesized as a larger precursor—proBDNF that is stored either in dendrites or in axons and is released in response to neuronal activity. Maturation of proBDNF to BDNF can occur in the cell, but also in the extracellular environment [279]. Intriguingly, proBDNF and BDNF have opposite effects, proBDNF being involved in pro-apoptotic signals [280]. Both physiological aging and neurodegenerative pathologies such as AD and PD cause cognitive disorders, possibly because of critical changes in the brain metabolism as well as in synaptic plasticity. In all these conditions, reduced levels of brain BDNF have been described. Moreover, studies performed on post-mortem brain tissue of patients with psychiatric disorders such as schizophrenia (SZ), bipolar disorder (BD), and major depressive disorder (MDD) also evidenced lower BDNF levels in brain regions involved in mnemogenic processes, such as the hippocampus [281]. Recently, EVs of neuronal origin have been isolated from peripheral blood by using an immunoprecipitation protocol based on the use of antibodies directed against the neuronal cell adhesion molecule L1CAM [250]. This approach allowed, for example, demonstration that older adults with walking speed decline have higher levels of proBDNF in EVs of neuronal origin [282]. Differences in the brain-derived EVs were also found in early AD patients, and in mice subjected to bilateral common carotid stenosis. The results of these experiments suggested, on one hand, that, as already mentioned, a pathological component of AD is cerebral hypoperfusion, and, on the other, that brain-derived EVs can be used as biomarkers to predict neural tissue alterations [273]. Interestingly, it has been also noticed that the levels of components of the amyloid β-peptide (Aβ)42-generating system in AD, such as β-site amyloid precursor protein-cleaving enzyme 1 (BACE-1), γ-secretase, soluble Aβ42, soluble amyloid precursor protein (sAPP)β, sAPPα, glial-derived neurotrophic factor (GDNF), and phosphorylated tau, are significantly higher in astrocyte-derived plasma exosomes than in neuron-derived exosomes. Moreover, all these proteins show levels significantly different in exosomes from patients when compared with exosomes from healthy controls [200].

On the other hand, changes in the miRNAs may be also critical as many of them target pathways involved in neuronal plasticity, learning, and memory acquisition, as well as mRNAs encoding neurotrophic factors such as BDNF itself [283]. Thus, identification and quantification of specific miRNAs present in circulating, brain-derived EVs can be of the most importance for diagnosis of brain cognition deficit. However, it is worth noting that this important opportunity is still partially hampered by the problems encountered in purification and analysis of EV-miRNAs [124,284], and of EVs themselves [285].

### 6.2. Natural as Well as Engineered EVs as Drug Carriers

Even more exciting is the potential of EVs as carriers for therapy. EVs represent, indeed, a system already tested by nature itself to deliver messages from one cell to various targets. Starting from their biological properties, with the help of suitable biotechnological techniques, it could be thus possible to manipulate them and obtain intelligent bullets to be loaded with therapeutic molecules, and to be specifically delivered to the desired target tissues. Moreover, given their compositional similarity with the cells of the body, EVs are almost non-immunogenic and can have an intrinsic specificity [286,287,288]; for example, it was reported that exosomes prepared from cultured cell lines were able to deliver curcumin, an anti-inflammatory agent, to activated mouse myeloid cells in vivo [289]. Notably, the exosomes determined on their own the target specificity [289]. 

In the context of the potential therapies aimed at targeting CNS pathologies, obviously of the most relevance is the EV’s ability to cross the BBB [136]. Other nanoparticles, also able to deliver their content to the brain, have been already tested, but it has been found that the carriers are often toxic and, anyway, rapidly cleared by the mononuclear phagocyte system [286].

One of the first interesting results in the field was the successful delivering to the mouse brain of a short interfering RNA (siRNA) by systemic injection of exosomes used as carriers; targeting was achieved by engineering the mouse dendritic cells to express an exosomal membrane protein, fused to a small peptide derived from the rabies virus glycoprotein (RVG). This peptide specifically targeted neuronal cells and BCECs and promoted BBB crossing [290]. More recently, in a xenotransplanted zebrafish (*Danio rerio*) model of brain cancer, exosomes, purified from brain cell lines, delivered anti-cancer drugs such as paclitaxel and doxorubicin to the tumour [291]. 

Similarly, the systemic treatment of a mouse model of PD with macrophages transfected with a plasmid DNA encoding the antioxidant enzyme catalase caused a significant increase of catalase concentration in the mouse brain. As a consequence, a reduction of inflammation and amelioration of PD conditions were observed [292]. From this study, it was also deduced that the transfected macrophages produced EVs containing active catalase as well as the corresponding mRNA and plasmid [293]. Starting from this observation, an exosome-based delivery system for catalase was developed to treat PD. In order to load catalase into the exosomes, a variety of methods were used such as incubation at room temperature, permeabilization with saponin, freeze–thaw cycles, and sonication. The loaded exosomes were readily taken up by neuronal cells in vitro. Moreover, a significant amount of exosomes was also detected in the PD mouse brain after exosome intranasal administration. The treatment provided significant neuroprotective effects both in vitro and in the PD model [293].

Interestingly, exosomes derived from a mouse neuroblastoma cell line or from human cerebrospinal fluid can counteract the synaptic-plasticity-disrupting activity of both synthetic and AD brain-derived Aβ peptide. The effect seems to be due to absorption of Aβ on the exosome surface (where it probably binds to the cellular prion protein PrPc) [294]. This finding is of interest because it suggests that one of the function of EVs might be to sequester the excess of Aβ, allowing its transport outside the brain, thus protecting neurons from the synapse plasticity impairments otherwise induced by the peptide. It has been also reported that neuronal exosomes are more suitable than glial exosomes in capturing Aβ because their membranes contain higher amounts of glycosphingolipids [295]. 

EVs have been also successfully used to mediate adeno-associated virus (AAV)-driven gene delivery into the murine retina [296]. These exosome-associated AAV vectors have been termed vexosomes (vector-exosomes) [297].

Perhaps one of the most relevant observations in the field of therapeutic applications of EVs concerns EVs derived from adult stem cells, such as the already mentioned MSCs. In general, MSCs have been considered very attractive for therapy, given their ability to promote tissue repair. If transplanted into a damaged tissue they are able indeed to secrete paracrine factors, which can ameliorate chronic-degenerative diseases [257,298]. In the brain, MSCs provide a suitable environment for axonal growth and neurogenesis, thus ameliorating neurological deficits [257]. Now, MSCs are also very active producers of EVs, and these EVs seem to share with intact MSCs the ability to counteract degenerative diseases and cognition deficits while promoting axonal growth, learning, and memory [299,300]. Moreover, they do not replicate and cannot undergo uncontrolled division after implantation. Finally, they are smaller than MSCs and their use in therapy is safer because of a decreased risk of thrombotic events [257]. Notably, by blocking the prostaglandin E2 receptor (EP4) with an EP4 antagonist, it is possible to increase release from MSCs of EVs with the same properties in vivo without the need to purify EVs to be used for the treatment [301].

As discussed in the previous paragraphs, the communication between oligodendrocytes and neurons, essential for axon myelination and for the survival and functionality of both cell types, is also largely mediated by EVs. We can thus suppose that EVs might also have a therapeutic role in pathologies characterized by demyelination, such as multiple sclerosis (MS). Moreover, also in this kind of pathology, MSC-derived exosomes have been shown to have a therapeutic effect by promoting oligodendrocyte differentiation and re-myelination in a model of subcortical ischemic stroke; these effects are probably due, at least in part, to their ability to deliver anti-inflammatory factors [302].

Since MSC-derived EVs are also able to ameliorate hippocampal synaptic impairment after ischemia [303] as well as memory dysfunction after status epilepticus [304], these vesicles are at the moment the object of much research aimed at finding out new therapies for brain injury and malfunction. 

Many of the described therapeutic properties of MSC-derived EVs, like their physiological activities, depend on the molecules they deliver. Among the proteins transported by MSC-derived exosomes some are of particular interest; for example, it has been found that they contain: neprilysin (an enzyme that catalyzes the proteolysis of the Aβ peptide), Sirtuin-1 (SIRT1, a deacetylating enzyme that targets chromatin proteins and transcription factors), Wnt3a (a protein able to activate the β-catenin signalling pathway), ephrins (proteins involved in axon guidance and synapse formation during development), and prosaposin (PSAP, a putative neurotrophic factor that stimulates maturation of hippocampal neurons), together with nerve growth factor (NGF) and BDNF (for a more comprehensive list of proteins found in these exosomes, see Table 1 in [257]). Beside proteins, MSC-derived exosomes also contain many miRNAs such as those belonging to the so called miR-17-92 cluster (miR-17, miR-18a, miR-19a, miR-19b, miR-20a, and miR-92a) involved in neurite remodelling and neurogenesis [232,233]. 

Interestingly, exosomes derived from hypoxia-preconditioned MSCs can rescue synaptic dysfunction and cognitive decline in an animal model of early-onset AD (APP/PS1 mice, which are double transgenic mice expressing a chimeric mouse/human APP, and a mutant human presenilin 1, PS1) [242]. This effect seems to be due to miR-21, which indeed significantly increases in MSCs (and exosomes) after hypoxic treatment [242].

### 6.3. Modulation of EV Release by Chemical Compounds

It is finally worth noting that a further therapeutic approach could be based on the ability of specific compounds to modulate release of EVs from tissues. For example, through the screening of a large collection of compounds, a potent inhibitor of the neutral sphingomyelinase 2 (nSMase 2) has been recently identified; nSMase 2 is an important regulator of EV biogenesis and its inhibition has been suggested to be useful in some neurological disorders. The compound (2,6-Dimethoxy-4-(5-Phenyl-4-Thiophen-2-yl-1*H*-Imidazol-2-yl)-Phenol (DPTIP) showed both an excellent pharmacokinetic profile and the ability to cross the BBB [305]. DPTIP blocked in a dose-dependent manner EV release from primary astrocytes and inhibited brain inflammation. Phenyl(*R*)-(1-(3-(3,4-dimethoxyphenyl)-2,6-dimethylimidazo[1,2-b]-pyridazin-8-yl)pyrrolidin-3-yl)-carbamate (PDDC) is a second and potent, non-competitive inhibitor of nSMase 2. PDDC is both brain penetrant and orally available [306]. Interestingly, inhibition of nSMase2 also decreases the transfer of oligomeric aggregates of α-synuclein into exosomes and the resulting spreading between neurons, thus suggesting that the inhibitors of nSMase2 might be used as new kinds of drugs for the therapy of PD [307].

## 7. Conclusions and Perspectives

The causal relationship between learning/memory and the ability of the neural circuits to undergo long-lasting, adaptive modifications of the strength and efficacy in the connections between neurons has been now studied for almost 50 years. In spite of the great interest and the huge efforts in the field, the cellular and molecular mechanisms that allow functioning of the amazing machine called the brain are still not completely understood. It is clear, however, that they rely on the continuous crosstalk between neurons and the non-neuronal cells around them. Astrocytes, in particular, have crucial functions in the formation and potentiation of the neural circuits. While these relatively new acquisitions are now being used even to implement computational systems neuroscience [308], the acknowledgement that both neurons and glial cells release EVs of different kinds, both in physiologic and pathological conditions, is giving us the possibility to explore new routes through which these cells can communicate over long distances with each other, as well as with the rest of the organism.

Moreover, the ability of EVs, and especially of exosomes, to cross the BBB in both directions is opening the way to use them both in diagnosis [250,273] and therapy [251,287,288]. As discussed, indeed, composition (as well as the amount) of brain-derived circulating EVs can change in brain disorders and in neurodegenerative diseases; in addition, a variety of methods have been developed to load drug molecules of different sizes into them.

We have, however, to remember that the successful use of EVs in diagnosis depends on the efficacy of the protocols used for their purification from the body fluids and for the analysis of their components; all these steps actually still present some challenges [124,284,285]. In spite of the clear advantages, the use of EVs as drug carriers presents even more challenges because of the fact that EVs are heterogeneous structures that contain hundreds of different proteins (among which are membrane ligands and receptors) and a complex set of both coding and non-coding RNAs, for many of which the functions are not yet clear, and which differ depending on the specific cellular ancestry, as well as the actual physiologic state of the cells [251]. Moreover, in many experiments, in order to obtain high amounts of exosomes, rapidly growing cell lines have been used that might harbour genetic and/or epigenetic modifications leading to secretion of EVs with main/side effects different from the expected ones.

In conclusion, EVs offer a potent tool for studying and explaining at least some of the cellular and molecular mechanisms underlying learning and memory, as well as the alterations found in neurodegenerative and psychiatric disorders. Moreover, they have been also used as circulating biomarkers and represent a powerful tool for drug delivery to the brain, provided that, starting from the already obtained promising results, a further effort is done for guaranteeing at best their safety.

## Figures and Tables

**Table 1:** Examples of miRNAs probably involved in learning/memory processes. References indicating their presence in extracellular vesicles are given in the last column.

miRNA [Refs]	Targets [Refs]	Primary Functions [Refs]	Presence in Exosomes/MVs [Refs]
miR-9-derived miR-9-3p [226]	Dystrophin [226]; Voltage-dependent Calcium channel, g subunit [226,227]; Leucine rich repeat transmembrane neuronal 1 [226,228]; Cadherin 2 [226,229]; Fibronectin [230]; Calcineurin B, type I [226,231]	Regulates synaptic plasticity and memory [226]	Found in serum exosomes of acute ischemic stroke patients [217]
**miR-17-92 cluster**: miR-17, miR-18a, miR-19a, miR-19b, miR-20a, and miR-92a [213,232,233,234]	Phosphatase and Tensin Homolog (PTEN) [211,234]	-Regulate axonal outgrowth in development [211]; -Regulate adult hippocampal neurogenesis, anxiety, and depression [232]; -Enhance neuroplasticity and functional recovery after stroke [233,234]; -Enhance neurite remodeling, neurogenesis and angiogenesis in post-stroke rats [233]; -Ablation in mouse impairs hippocampal-dependent learning and memory [212]	miR-19a found in exosomes [213,233]
miR-26a [209]	PTEN, GSK-3, BDNF [209]	Stimulates neurite/axonal elongation [209]	Found in astrocytic exosomes [209]
miR-29c [235]	Beta secretase 1 (BACE1) [236]	This microRNA can be an endogenous regulator of the BACE 1 enzyme, and thus of beta amyloid precursor protein (APP) metabolism. [236]	Found in exosomes contained in frozen post-mortem prefrontal cortex from bipolar individuals [235]
miR-34a [237]	Activity-regulated cytoskeleton-associated protein (Arc) Chicken ovalbumin upstream promoter transcription factor-interacting proteins 2 (Ctip2) Transcription factor 4 (TCF4) Ubiquitin-conjugating enzyme E2 G1 (Ube2g1) [237]	Regulates Synaptic Efficacy in the Adult Dentate Gyrus In Vivo [237]; Overexpressed in Alzheimer’s Disease (AD) patients [238]	Found in exosomes of overexpressing primary neurons in culture [238]
miR-124 [239]	Targets, EZH2 [216] and the glucocorticoid receptor [240]	Regulate early memory phases [239]; miR-124 plays also a role in depression [240]; miR-124 regulates cell fate (prevents astrocytes from expressing neuronal proteins) [216]	miR-124 found in serum exosomes of acute ischemic stroke patients [217]
miR-125b [241,242]	Nestin [241]	Regulates differentiation and migration in neural stem/progenitor cells [241]	Found in exosomes from adult astrocytes [243]
miR-132 [244]	Eukaryotic elongation factor 2 kinase (eef2k) [244]	Regulates maintenance of brain vascular integrity [244]	Found in neuronal exosomes delivered to BCECs [244]
miR-134 [245]	Caspase-8 [245]	-Regulates survival of oligodendrocytes [245]; -Regulates the size of dendritic spines, excitatory synaptic transmission, and synaptic plasticity [246]	Found in bone marrow-derived mesenchymal stem cells (BMSCs) Exosomes [245]
miR-139 [247]	Cannabinoid receptor type 2 (CB2) [247]	Regulates hippocampal function, and affects learning and memory acquisition [247]	miR-139-derived miR-139-5p Found in exosomes of AD patients [248]
miR-497 [235]	B-cell lymphoma 2 (Bcl-2)/Bcl-w genes [249]	Regulates ischemic neuronal death in N2A neuroblastoma cells and also in a mouse model of middle cerebral artery occlusion (MCAO) [249]	Found in exosomes contained in frozen post-mortem prefrontal cortex from schizophrenia patients [235]
